# Future temperature-related excess mortality under climate change and population aging scenarios in Canada

**DOI:** 10.17269/s41997-023-00782-5

**Published:** 2023-06-12

**Authors:** Christopher Hebbern, Pierre Gosselin, Kai Chen, Hong Chen, Sabit Cakmak, Melissa MacDonald, Jonathan Chagnon, Patrice Dion, Laurent Martel, Eric Lavigne

**Affiliations:** 1 Climate Change & Innovation Bureau, Health Canada, Ottawa, ON Canada; 2 Institut National de La Recherche Scientifique (Centre Eau-Terre-Environnement), Québec, QC Canada; 3 Institut National de Santé Publique du Québec, Québec, QC Canada; 4 Department of Environmental Health Sciences, Yale School of Public Health, New Haven, CT USA; 5 Yale Center On Climate Change and Health, Yale School of Public Health, New Haven, CT USA; 6 Population Studies Division, Health Canada, Ottawa, ON Canada; 7 Meteorological Service of Canada, Environment and Climate Change Canada, Gatineau, QC Canada; 8 Centre for Demography, Statistics Canada, Ottawa, ON Canada; 9 School of Epidemiology and Public Health, University of Ottawa, Ottawa, ON Canada

**Keywords:** Climate change, Mortality, Projection, Temperature, Long-term, Population aging, Changement climatique, mortalité, projection, température, long terme, vieillissement de la population

## Abstract

**Objective:**

Climate change is expected to increase global temperatures. How temperature-related mortality risk will change is not completely understood, and how future demographic changes will affect temperature-related mortality needs to be clarified. We evaluate temperature-related mortality across Canada until 2099, accounting for age groups and scenarios of population growth.

**Methods:**

We used daily counts of non-accidental mortality for 2000 to 2015 for all 111 health regions across Canada, incorporating in the study both urban and rural areas. A two-part time series analysis was used to estimate associations between mean daily temperatures and mortality. First, current and future daily mean temperature time series simulations were developed from Coupled Model Inter-Comparison Project 6 (CMIP6) climate model ensembles from past and projected climate change scenarios under Shared Socioeconomic Pathways (SSPs). Next, excess mortality due to heat and cold and the net difference were projected to 2099, also accounting for different regional and population aging scenarios.

**Results:**

For 2000 to 2015, we identified 3,343,311 non-accidental deaths. On average, a net increase of 17.31% (95% eCI: 13.99, 20.62) in temperature-related excess mortality under a higher greenhouse gas emission scenario is expected for Canada in 2090–2099, which represents a greater burden than a scenario that assumed strong levels of greenhouse gas mitigation policies (net increase of 3.29%; 95% eCI: 1.41, 5.17). The highest net increase was observed among people aged 65 and over, and the largest increases in both net and heat- and cold-related mortality were observed in population scenarios that incorporated the highest rates of aging.

**Conclusion:**

Canada may expect net increases in temperature-related mortality under a higher emissions climate change scenario, compared to one assuming sustainable development. Urgent action is needed to mitigate future climate change impacts.

**Supplementary Information:**

The online version contains supplementary material available at 10.17269/s41997-023-00782-5.

## Résumé

**Objectif:**

Les changements climatiques devraient accroître les températures mondiales. La façon dont le risque de mortalité lié à la température évoluera n’est pas entièrement comprise, et la façon dont les changements démographiques futurs influeront sur la mortalité liée à la température doit être clarifiée. Nous étudions la mortalité liée à la température au Canada jusqu’en 2099, en tenant compte des groupes d’âge et des scénarios de croissance démographique.

**Méthodes:**

Nous avons utilisé les nombres quotidiens de mortalité non accidentelle pour 2000 à 2015 pour toutes les 111 régions socio sanitaires du Canada, en intégrant dans l’étude des régions urbaines et rurales. Une analyse en séries chronologiques en deux parties a été utilisée pour estimer les associations entre les températures quotidiennes moyennes et la mortalité. Premièrement, des simulations de séries chronologiques de températures moyennes quotidiennes actuelles et futures ont été élaborées à partir d’ensembles de modèles climatiques du Projet de comparaison croisée 6 (CMIP6) du modèle couplé à partir de scénarios de changements climatiques passés et projetés dans le cadre de voies socioéconomiques partagées (SSP). Ensuite, la surmortalité due à la chaleur et au froid et la différence nette ont été projetées jusqu’en 2099, ce qui tient également compte de différents scénarios régionaux et de vieillissement de la population.

**Résultats:**

De 2000 à 2015, nous avons recensé 3 343 311 décès non accidentels. En moyenne, une augmentation nette de 17,31% (eCI à 95%: 13,99, 20,62) de la mortalité excessive liée à la température dans le cadre d’un scénario d’émissions de gaz à effet de serre plus élevées est prévue pour le Canada en 2090–2099, ce qui représente un fardeau plus lourd qu’un scénario qui suppose des niveaux élevés de politiques d’atténuation des émissions de gaz (augmentation nette de 3,29%; eCI à 95%: 1,41, 5,17). La plus forte augmentation nette a été observée chez les personnes de 65 ans ou plus, et les plus fortes augmentations de la mortalité nette, de mortalité liée à la chaleur et au froid ont été observées dans les scénarios de population qui comprenaient les taux de vieillissement les plus élevés.

**Conclusion:**

Le Canada pourrait s’attendre à des augmentations nettes de la mortalité liée à la température dans le cadre d’un scénario de changement climatique à émissions plus élevées, comparativement à un scénario de développement durable. Des mesures urgentes sont nécessaires pour atténuer les répercussions futures des changements climatiques.

## Introduction

Climate change represents a leading global health threat for the twenty-first century (Watts et al., 2018). In Canada, surface temperatures have risen by 1.7 °C since 1948 (Bush & Lemmen, 2019) and are expected to increase about a further 5.44 °C in major cities towards the end of the century under the higher greenhouse gas emission scenario (Lee et al., 2019).

Sub-optimal temperature exposures are associated with a range of conditions that impact health, directly and indirectly. Temperature stress can have an indirect relationship with a variety of mortality causes, beyond hypo- and hyperthermia, and may exacerbate underlying conditions and result in increased mortality (Ebi et al., 2021). Studies have projected health impacts associated with changes in ambient temperature for the next century, predicting an increase in heat-related mortality (Gasparrini et al., 2017; Guo et al., 2018; Martínez-Solanas et al., 2021; Wang et al., 2022; Weinberger et al., 2018). However, most studies have focused mainly on major cities or urban areas and therefore have yet to incorporate temperature-health projections for rural and northern latitudes. Therefore, mortality projections are often based on estimates from major cities, ignoring possible urban–rural differences which may result in incorrect estimations (Chen et al., 2016; Hu et al., 2019). In addition, debates remain as to whether an increase in heat-related health impacts would be offset by decreases in cold-related health consequences. Furthermore, changing demographics and population aging are likely to impact climate change and health burdens. The elderly are particularly at risk from non-optimal temperatures (Benmarhnia et al., 2015), and accounting for population changes, in particular by age subgroups at fine geographical spatial resolution, will improve understanding of future risks (Chen et al., 2020; Harper et al., 2021; Xing et al., 2022). Establishing local baseline values of temperature-related excess mortality and projections under climate change and population growth scenarios across Canada are important for public health officials when planning and managing health services.

In this study, we estimate and project the impacts of climate change on temperature-attributable mortality across health regions in Canada, under alternative scenarios of global warming and different population growth scenarios.

## Materials and methods

### Study design

We used a two-stage time series analysis, as previously described (Gasparrini et al., 2017; Vicedo-Cabrera et al., 2019). We evaluated future associations between ambient temperature and daily counts of mortality in all 111 health regions across Canada, incorporating both urban and rural areas (Stieb et al., 2019). In Canada, health services are primarily under provincial responsibility, and health regions mostly correspond to the areas administered by public health departments or local authorities. The analysis was also carried out nationally. We acquired daily mean temperature time series, both observed from monitoring stations during the historical period and modeled according to climate change scenarios from the Shared Socioeconomic Pathways (SSPs), which were combined with the Representative Concentration Pathways (RCPs) (Gidden et al., 2019). These climate change scenarios are further defined below.

### Data sources and scenario models

We used the Vital Statistics Deaths Database from Statistics Canada in order to obtain daily mortality counts for all 111 health regions. We obtained the daily counts of non-accidental mortality (International Classification of Diseases 10th revision [ICD-10] codes A00–R99), cardiovascular mortality (ICD-10 codes I00–I99), and respiratory mortality (ICD-10 codes J00–J99) between 2000 and 2015.

Daily mean temperatures from 2000 to 2015 were obtained from Environment and Climate Change Canada, from a highly dense network of 8305 weather stations; a daily mean temperature, the 24-h average from hourly measurements, was used to represent each health region’s exposure. We averaged temperature observations across all weather stations within each health region to represent the health region’s daily mean temperature exposure. This index can be readily interpreted for decision-making purposes and has previously been used in health projection studies (Guo et al., 2014).

For each health region, socioeconomic and demographic data were obtained from Statistics Canada and the Canadian Community Health Surveys (CCHS) that occurred from 2000 to 2015. The CCHS is a cross-sectional survey that gathers data on health status, health care use, and determinants of health for the Canadian population (Statistics Canada, 2023). The health region–level data included the proportions of the Canadian population for 2000 to 2015 in the following categories: (1) proportion under 65 years of age and proportion 65 years and over; (2) individuals with cardiovascular disease, respiratory disease, or diabetes; (3) outdoor workers; (4) adults with less than a high school diploma, used as an indicator of lower SES; and (5) the proportion within each health region residing in urban areas. The urbanization level, expressed as percent of the health region considered urban, was determined using Statistics Canada data that identified the respondent’s residence based on census geography. Areas are classified by Statistics Canada as urban population centres when the population is 1000 or more, and population density is at or greater than 400 per square kilometre. Large population centres have a population of 100,000 or more.

**Fig. 1:**
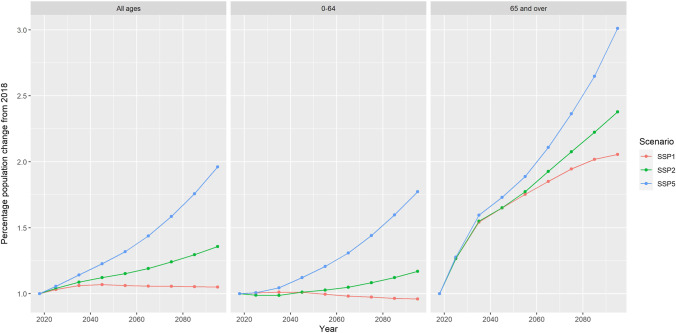
Mean projected percentage change for all health regions, from the 2018 baseline, by population growth scenarios

Population projection scenarios were provided by Statistics Canada at the health region level, developed to be consistent with the 2018 National Population Projections that had been previously developed at Provincial and Territorial geographic scales. Using a bottom-up, cohort-component model, three distinct scenarios were developed based on different assumptions of rates of fertility, mortality, immigration, emigration, non-permanent residents, and internal migration (KC & Lutz, 2017), and that also correspond to the SSPs that are used in climate modeling. Here, the population growth scenario SSP1 represents medium migration and fertility rates, and low mortality; SSP2 represents medium growth in fertility, mortality, immigration, emigration, non-permanent residents, and internal migration; and a high growth scenario, SSP5, assumes high fertility, low mortality, high immigration, and high rates of non-permanent residents. The assumptions used for each scenario are summarized in Supplementary Table S1, and the percentage-projected change for each scenario is shown in Fig. 1.

Future impacts of temperature-related mortality were evaluated for three climate change scenarios, using modeled climate and mortality projections. The climate change scenarios were defined as SSP*x*-*y*, where *x* is a specific SSP of future climate adaptation and mitigation policies, and *y* is a specific RCP of different radiative forcings of the global atmosphere. The different RCPs for average radiative forcing at the top of the global atmosphere, at the year 2100, are projected to be 2.6 W/m^2^ (for RCP2.6), 4.5 W/m^2^ (RCP4.5), 7.0 W/m^2^ (RCP7.0), and 8.5 W/m^2^ (RCP8.5). The combination of both describes the future temperature trajectory for the twenty-first century under the climate adaptation and mitigation policy of a specific SSP in achieving a RCP objective.

Three climate scenarios with different climate adaptation and mitigation policies combined with different radiative forcings were extracted, namely SSP1-2.6 (i.e., representing sustainability/taking a green road pathway, where CO_2_ emissions are cut and reach net zero after 2050 and temperatures reach around 1.8 °C higher by 2100), SSP2-4.5 (i.e., representing a lower emission and “middle-of-road scenario,” where CO_2_ emissions do not reach net zero by 2100 and temperatures rise by 2.7 °C by the end of the century), and SSP5-8.5 (i.e., fossil-fueled development scenario, which sees CO_2_ emissions double by mid-century and average global temperatures will be 4.4 °C higher) (Zhao et al., 2021). The selection of these SSPs/climate change scenarios was based on the availability of regional population projections in Canada and merged to obtain future estimates of excess mortality that could be attributed to temperature. Time series of daily mean temperatures were acquired for the three climate change SSPs (SSP1-2.6, SSP2-4.5, and SSP5-8.5). Future projected temperature series were generated for each SSP using 27 general circulation models (GCMs) made available by the Coupled Model Inter-Comparison Project 6 (CMIP6) (Zhao et al., 2021). The CMIP6 database provides daily mean temperature time series for historical (1973–2014) and projected (2015–2100) periods, downscaled to 0.25° × 0.25° spatial resolution. We obtained the projected daily temperature series for each health region for the period 2015–2099. Deviations between projected and observed daily temperature series may bias results in the impact projections, so we applied a bias-correction method to recalibrate the projected daily temperature series (Hempel et al., 2013). We projected the daily mortality time series for the period from 2015 to 2099 using the average observed daily mortality counts from 2000 to 2015 (Gasparrini et al., 2017; Vicedo-Cabrera et al., 2019).

### Statistical analysis

We employed a two-stage time series analysis to estimate temperature-mortality associations, as described previously (Gasparrini et al., 2017; Vicedo-Cabrera et al., 2019). Briefly, in the first stage, we obtained health region–specific estimates of the associations between temperature and mortality outcomes through quasi-Poisson regression, controlling for season, long-term trends, and day-of-the-week (Gasparrini et al., 2015). We modeled the associations between temperature and mortality outcomes with a distributed lag non-linear model, applying a bidimensional cross-basis spline function with 21 days of lag (Gasparrini et al., 2017; Vicedo-Cabrera et al., 2019). We employed a natural cubic spline to model daily temperature with three internal knots at the 10th, 75th, and 90th percentile of the health region–specific temperature distributions. A natural cubic spline with three internal knots, equally spaced in the log-scale, was used for the lag dimension. Model selection for temperature-mortality and lag-response functions was based on the best model fit using the Akaike Information Criteria (AIC) as well as visual inspections of preliminary findings. We therefore conducted sensitivity analyses using different knot specifications and degrees of freedom.

In the second stage, we pooled the health region–specific estimates using multivariate meta-regression models to obtain non-linear temperature and mortality associations for Canada overall (Gasparrini et al., 2017; Sera et al., 2019). In order to capture part of the heterogeneity across health regions, we included a number of meta-predictors such as health region–specific proportions of the 2000–2015 Canadian population for these groups: elderly (≥ 65 years of age); individuals with cardiovascular disease, respiratory disease, or diabetes; adults with less than a high school diploma; outdoor workers; and the proportion of the population within each health region residing in urban areas. We also captured as meta-predictors indicators for climate classification (Cakmak et al., 2018), mean temperature, and temperature range. We then derived the best linear unbiased prediction of the overall cumulative associations between temperature and mortality outcomes in each health region (Gasparrini & Armstrong, 2013).

Following the first two stages, we then projected excess mortality due to temperature using the modeled daily series of temperature and mortality assuming no adaptation or population changes in the initial model, similar to previous studies (Gasparrini et al., 2017; Onozuka et al., 2019; Sera et al., 2019). Briefly, for each health region, the attributable deaths and attributable fraction were calculated using the overall cumulative relative risk that corresponded to each day’s temperature. The minimum mortality temperature (the optimal temperature) was used as the reference. The total excess mortality attributable to non-optimal temperature was obtained from the sum of the contributions from all days of the time series; the cold- and heat-attributable components were identified by summing the subsets of days where temperatures were below or above the optimal temperature (Gasparrini et al., 2017; Onozuka et al., 2019; Sera et al., 2019). For each population growth scenario, the difference between the 2018 baseline and the mid-decadal population projection for each health region and age group (all ages, under 65, and 65 and over) was used to adjust the projected mortality time series.

The excess mortality was first calculated separately for each health region and each combination of GCMs and SSPs. The attributable fractions were computed as GCM-ensemble averages by aggregating by decade and SSP. The related total number of deaths was used as the denominator. We used a Monte Carlo approach (5000 simulations) to obtain empirical confidence intervals (eCIs), to quantify uncertainty in both the estimation of the exposure–lag–response relationships and in the climate projections for the GCMs (Gasparrini et al., 2017; Sera et al., 2019). We present percent excess deaths attributable to both heat and cold across the three different SSP scenarios for the current period (i.e., 2010–2019) and for each decade of the twenty-first century. We also present the net difference (i.e., combining heat and cold contributions) in percent excess mortality for future decades compared to the baseline decade (i.e., 2010–2019). Finally, we present projections based on health region–level characteristics (i.e., socioeconomic and demographic meta-predictors), assuming those are held constant in the future. We evaluated whether those health region–level characteristics modified the projected net differences. A Wald test was used in meta-regression models to test for effect modification. All analyses were conducted with R (version 3.5.3), using the packages dlnm and mixmeta.

## Results

A total of 3,343,311 non-accidental deaths were identified between 1 January 2000 and 31 December 2015, with an average of 5.6 deaths per day in 111 health regions across Canada (Table 1). The daily mean temperature was 4.9 °C, ranging from − 43.6 to 31.9 °C. The yearly average number of deaths by health region is shown in Supplementary Table S2, and ranged from 7.8 in Athabasca Health Authority to 15,090 in Région de Montréal.

**Table 1:** Descriptive statistics of health region–level variables in Canada (*n* = 111)

	Mean	Range (min–max)
Daily temperature, 2000–2015 (°C)	4.9	− 43.6 to 31.9
Projected increase in temperature (°C) (2090–2099 vs. 2010–2019)		
SSP1-2.6	0.9	0.5–1.3
SSP2-4.5	2.6	2.0–3.5
SSP5-8.5	5.6	4.5–7.7
Sum of daily deaths, 2000–2015		
Non-accidental	5.6	0–143
All cardiovascular	1.7	0–52
All respiratory	0.5	0–24

**Fig. 2:**
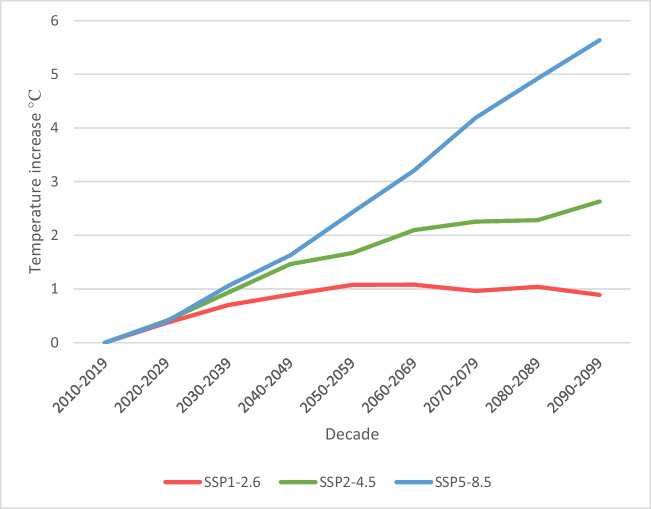
Decadal temperature trends by scenario in Canada compared to the 2010–2019 decade

The projected increase in average daily temperature is expected to be 5.6 °C (range: 4.5–7.7) under a high-end emission scenario (SSP5-8.5) by the last decade of the twenty-first century compared to 2010–2019 (Table 1; Fig. 2). However, this increase is expected to be lower under climate change scenarios that assume a sustainability pathway (SSP1-2.6). For instance, an average temperature increase of 0.9 °C (range: 0.5–1.3) is expected under SSP1-2.6. Projected temperature increases for each health region are presented in Supplementary Table S3 and Supplementary Figure S1; it appears that health regions located in northernmost sectors will experience more dramatic increases in temperature, for example, up to 7.7 °C in Nunavut by 2090–2099.

The projected trends in heat- and cold-related excess non-accidental mortality, for three SSPs in Canada, are summarized in Tables 2, 3, and 4. We also present in Supplementary Table S4 future impacts under an assumption of no population change. In general, there was a common trend of an increase in heat-related mortality across SSP scenarios, which resulted in a net increase in excess mortality. Steeper gradients in projections were observed for SSP5-8.5 while shallower trends were found under the scenario that assumed mitigation strategies (SSP2-4.5) and much smaller impacts for the sustainability pathway scenario (SSP1-2.6). For example, heat-related non-accidental mortality is projected to increase from 0.41% (95% eCI: 0.24, 0.59) in 2010–2019 to 10.74% (95% eCI: 8.02, 13.45) in 2090–2099 under SSP5-8.5 (Table 4). This results in a net increase in excess mortality of 17.31% (95% eCI: 13.99, 20.62) in 2090–2099 compared to the 2010–2019 decade. In contrast, heat-related excess non-accidental mortality is projected to increase from 0.68% (95% eCI: 0.33, 1.04) in 2010–2019 to 0.88% (95% eCI: 0.43, 1.33) in 2090–2099 under SSP1-2.6, resulting in a net increase of 3.29% (95% eCI: 1.41, 5.17) (Table 2). Overall, the net increase in excess mortality is expected to be higher in SSP2-4.5 (5.41%, 95% eCI: 3.64, 7.19) compared to SSP1-2.6, but projected impacts appear lower than SSP5-8.5. In addition, cold-related mortality impacts are expected to decrease under SSP1-2.6, but will increase under SSP2-4.5 and SSP5-8.5 (Tables 2, 3, and 4).

**Table 2:** Heat-related, cold-related, and net change in excess non-accidental mortality (%) with 95% empirical confidence interval (eCI) by period under SSP1-2.6 population aging and climate change scenarios in Canada

Age group	Effect	Period		
		2010–2019	2050–2059	2090–2099
All	Heat	0.68 (0.33–1.04)	0.97 (0.54–1.40)	0.88 (0.43–1.33)
	Cold	7.49 (2.54–12.43)	5.52 (0.53–10.51)	5.31 (0.11–10.50)
	Net	-	4.29 (2.50, 6.08)	3.29 (1.41, 5.17)
65 and over	Heat	1.34 (0.73–1.94)	1.33 (0.71–1.95)	1.27 (0.72–1.82)
	Cold	− 5.83 (− 31.77 to 20.11)	10.83 (3.32–18.35)	4.04 (− 9.00 to 17.09)
	Net	-	9.65 (5.25–14.05)	4.42 (− 5.18 to 14.03)
Under 65	Heat	0.20 (− 1.09 to 1.48)	0.004 (− 1.31 to 1.32)	0.13 (− 0.98 to 1.24)
	Cold	− 9.77 (− 22.18 to 2.65)	− 13.00 (− 25.82 to − 0.18)	− 9.08 (− 21.15 to 3.00)
	Net	-	− 5.85 (− 12.35 to 0.64)	− 2.23 (− 8.03 to 3.56)

**Table 3:** Heat-related, cold-related, and net change in excess non-accidental mortality (%) with 95% empirical confidence interval (eCI) by period under SSP2-4.5 population aging and climate change scenarios in Canada

Age group	Effect	Period		
		2010–2019	2050–2059	2090–2099
All	Heat	1.25 (0.6, 1.9)	0.94 (0.45, 1.43)	1.67 (1.08, 2.26)
	Cold	5.93 (0.88, 10.99)	5.92 (0.55, 11.29)	7.21 (2.45, 11.98)
	Net	-	4.35 (2.57, 6.13)	5.41 (3.64, 7.19)
65 and over	Heat	2.51 (1.51, 3.5)	2.28 (1.3, 3.27)	2.14 (1.32, 2.96)
	Cold	− 6.1 (− 33.11, 20.91)	11.07 (3.72, 18.43)	3.94 (− 9.45, 17.33)
	Net	-	10.89 (6.43, 15.36)	5.47 (− 4.94, 15.87)
Under 65	Heat	− 0.02 (− 2.03, 2)	− 0.33 (− 2.54, 1.88)	− 0.18 (− 1.86, 1.5)
	Cold	− 9.24 (− 21.22, 2.74)	− 12.72 (− 25.16, − 0.28)	− 8.74 (− 20.69, 3.22)
	Net	-	− 7.24 (− 14.19, − 0.3)	− 2.46 (− 8.18, 3.26)

**Table 4:** Heat-related, cold-related, and net change in excess non-accidental mortality (%) with 95% empirical confidence interval (eCI) by period under SSP5-8.5 population aging and climate change scenarios in Canada

Age group	Effect	Period		
		2010–2019	2050–2059	2090–2099
All	Heat	0.41 (0.24, 0.59)	2.97 (1.99, 3.95)	10.74 (8.02, 13.45)
	Cold	3.81 (1.08, 6.53)	7.4 (1.51, 13.29)	8.87 (2.39, 15.36)
	Net	-	7.33 (4.85, 9.82)	17.31 (13.99, 20.62)
65 and over	Heat	6.26 (3.85, 8.67)	4.96 (2.83, 7.09)	4.76 (2.91, 6.62)
	Cold	− 7.74 (− 37.49, 22.01)	11.51 (4.21, 18.82)	3.87 (− 9.88, 17.61)
	Net	-	14.51 (9.12, 19.9)	8.35 (− 3.18, 19.87)
Under 65	Heat	− 1.47 (− 6.06, 3.12)	− 2.36 (− 8.68, 3.95)	− 2.52 (− 7.34, 2.31)
	Cold	− 9.46 (− 21.88, 2.95)	− 8.83 (− 21.1, 3.45)	− 8.83 (− 21.1, 3.45)
	Net	-	− 12.96 (− 25.59, − 0.33)	− 9.27 (− 22.06, 3.51)

For those 65 and over, heat-related excess non-accidental mortality is projected to change from 6.26% (95% eCI: 3.85, 8.67) in 2010–2019 under SSP5-8.5 to 4.76% (95% eCI: 2.91, 6.62) in 2090–2099 (Table 4). This results in a net increase in excess mortality of 8.35% (95% eCI: − 3.18, 19.87) in 2090–2099 compared to the 2010–2019 decade. In comparison, heat-related excess non-accidental mortality is projected to change from 1.34% (95% eCI: 0.73, 1.94) in 2010–2019 under SSP1-2.6 to 1.27% (95% eCI: 0.72, 1.82) in 2090–2099 (Table 2). Estimates of heat-related excess non-accidental mortality are negative in the younger age group, while they remain positive in those aged 65 and over.

**Fig. 3:**
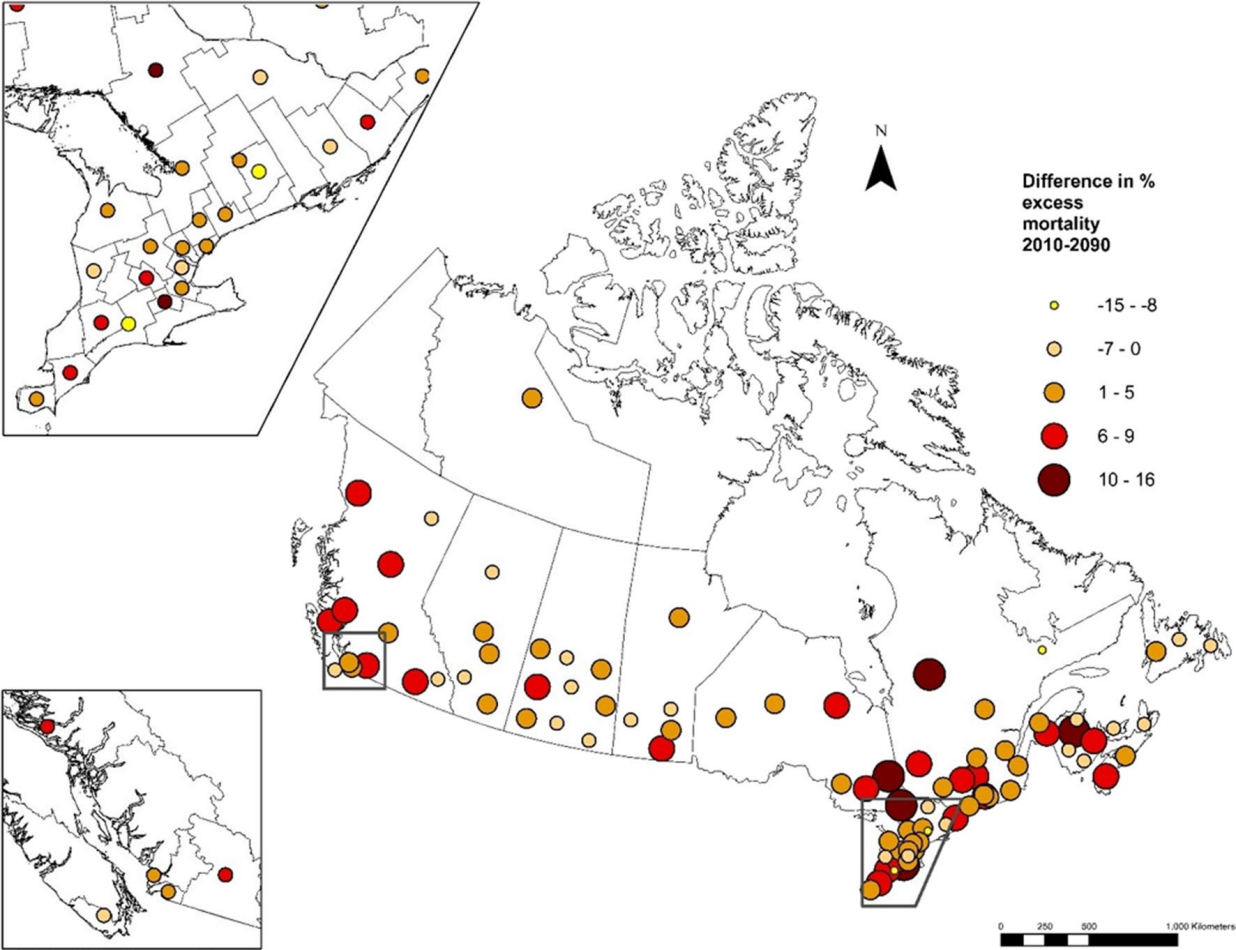
Projected net change in excess mortality (%) in 2090–2099 compared to 2010–2019 under SSP5-8.5 in selected health regions with more stable estimates

The respective data across each health region under two climate change scenarios are shown in Supplementary Table S6. For instance, a few health regions in the Maritimes, the Prairies, and some northern locations showed a net decrease in excess mortality under the same scenarios. To illustrate these geographical differences, projected net excess mortality (%) in 2090–2099 compared to 2010–2019 under SSP5-8.5 is mapped in Fig. 3, and ranged from − 15% to 16% in heath regions with stable estimates (i.e., including only health regions for which the standard error is less than 5 times the calculated estimate).

In sensitivity analyses, we found that the historical temperature-mortality associations had an optimal model fit when using three internal knots at the 10th, 75th, and 90th percentile of the health region–specific temperature distributions and three internal knots in the lag dimension (based on the minimal value of AIC) (Supplementary Table S5).

Projections of heat- and cold-related excess cardiovascular and respiratory mortality lacked statistical power for proper interpretation and therefore could not be presented in whole in this paper. The results for the health-level characteristics—urbanization level, percentage elderly population, percentage of the population with comorbidities, the percentage that are outdoor workers, and the percentage with less than a high school education—showed in general no statistically significant effect modification (Supplementary Table S7). These findings might be related to the lack of statistical power to fully evaluate effect modifiers for health-level characteristics.

## Discussion

We estimated future projections of temperature-related excess mortality across Canada according to different climate change scenarios. We found a net increase in temperature-related excess mortality overall for Canada, under a higher emission scenario, while accounting for population aging. We also found that the elderly will likely suffer to a greater extent from future temperature increases. Our findings indicate that climate change may increase the burden on mortality in Canada in the future, and that this burden shows important spatial variations of interest at the regional and local levels, as well as notable differences under various population scenarios and age groups.

This study showed that, under a higher emission scenario, climate change may increase heat-related excess mortality, and that will not be balanced by a decrease in cold-related deaths. As a result, there may be an overall positive net increase in mortality for Canada. These findings agree with a study that included worldwide projections (Gasparrini et al., 2017), including 26 cities across Canada, although the projected net increase (1.9% net difference, 95% eCI: 0.6 to 4.8) was lower than that identified in the current report (17.31% net difference, 95% eCI: 13.99 to 20.62) in which we accounted for population changes at regional levels. Another investigation for 15 cities in Canada showed that the net increase in mortality may only be observed in some cities (i.e., Hamilton, London, Montreal, and Regina) (Martin et al., 2012).

Few studies on future temperature-related mortality accounted for population aging or performed age-stratified analyses using age-specific temperature-mortality relationships, in particular at sub-national or regional levels (Chen et al., 2020). Here, we extend previous analyses by more accurately accounting for population aging. A recent review of studies on future temperature-related mortality under climate change found that accounting for population aging amplified the projected mortality burden of increases in temperature (Chen et al., 2020). A recent study by Xing et al. found that projections of future temperature-related cardiovascular mortality in Beijing, China, would be amplified by population aging, even after accounting for improvements in urbanization (Xing et al., 2022). In our study, we did not account for urbanization improvements over future decades, which is a limitation. In fact, improvements in urbanization over time have been found to be associated with lower temperature-related mortality risk (Xing et al., 2020), partly due to better access to medical and health resources as well as higher disposable income and higher levels of education (Chen et al., 2016). Overall, the resulting increased burden of mortality that we found highlights the importance of including age-specific population projections and age-specific temperature-mortality relationships into the analyses.

Our study also showed that temperature-related excess mortality depends on climate change scenarios of SSPs based on greenhouse gas emission scenarios. The greatest increase in the net difference in mortality was found under a scenario characterized by unabated greenhouse gas emissions, while there were only slight increases in the net difference in excess mortality under a climate change scenario assuming mitigation strategies and sustainability. Therefore, our findings have important implications for climate policy makers and public health officials in developing strategies to contain global warming and prevent adverse health impacts.

Understanding the future trajectory of temperature-related mortality under climate change is improved by accounting for health region–specific population change, with the geographic specificity representing an advance over using previously available national-, provincial-, and territorial-level projections (Chen et al., 2020). The different population change scenarios used indicated a wide difference in projected mortality rates. The scenarios that incorporated the greatest degree of aging indicated the largest increases in both net and heat- and cold-related mortality. Impacts were also greater in those aged 65 and over, consistent with recent research (Chen et al., 2020).

In a qualitative analysis of local health authority needs for adapting to the impacts of climate change, Austin et al. (2019) identified the estimation or quantification of the disease burden of climate change on health as a priority. Here we provide quantitative information that can be used at the local health authority level for climate change adaptation planning, as well as at the national level to project climate change risks for Canada as a whole. These climate change and health risk estimates and projections are also a key step in the Building Resilience Against Climate Effects (BRACE) framework, developed to facilitate public health adaptation to climate change (Marinucci et al., 2014).

### Limitations of the study

Some limitations must be acknowledged. We did not account for potential population adaption to future temperatures (Arbuthnott et al., 2016); our approach allowed us to explore the impacts of different climate change and population growth scenarios on projected mortality risk, but relies on the assumption that the relationship between temperature and mortality will be stable over time. As Canada is getting older, demographic trends could increase populations at higher risk for such impacts, while mitigation and adaptation could decrease these risks, and modify the temperature-mortality relationship over the long-time horizon that we used. In addition, we also did not account for potential changes in urbanization, as mentioned above (Xing et al., 2020). Adaptation could also occur through improved indoor temperature regulation such as air conditioning, the prevalence of which can be expected to change, although the attenuation of heat-related mortality risk might be limited: Sera et al. (2020) found that in Canada, in a study of 311 regions, increased air conditioning could explain 16.7% of the observed reduction in excess deaths due to heat that were seen over the 5-year study period. Therefore, our findings should not be viewed as predictions of future excess mortality, but rather possible projected impacts under hypothetical scenarios. Considerable uncertainty is also present because of variability in climate modeling, the long-time scales considered in this study, and imprecise exposure–response estimates, which may have affected our future projections of temperature-related excess mortality, notably those related to the net impact. There could also be misclassification bias of exposure to temperature for the historical period since we used ground weather stations to represent population exposure. We also relied on average temperature increases rather than investigating other temperature metrics (e.g., daily maximum temperature, diurnal temperature range, heat wave) and therefore, our findings cannot be generalized to all the different future variations of exposure to temperature changes. Our reported estimates by age groups could also be limited due to lack of statistical power and, therefore, should be interpreted cautiously. Finally, we did not have information on individuals with prior comorbidities which may also affect future projections; however, the aggregated information on such morbidities was available at the health region level for inclusion in the model.

## Conclusion

Our study shows that Canada will experience an increase in temperature-related mortality under higher greenhouse gas emission scenarios through to 2099, that the impacts are greatest in those aged 65 and over, and that accounting for population aging reveals important differences in mortality projections. These findings should be informative for developing public health policies at the health authority scale, and strategies for climate change mitigation and adaptation at both national and local levels. Future research should try to incorporate more information on adaptation measures implemented over time as well as more precise susceptibility variables of exposed groups in health regions, in order to adjust future simulations.

## Contributions to knowledge

What does this study add to existing knowledge?

- Few studies in Canada have examined whether temperature-related impacts are expected to increase with climate change. In this population-scale study, we consider changing demographics, population aging, and different climate model scenarios, to project future climate-related health risks due to climate change.
- This study was conducted at the administrative health region geographic scale, so temperature-related mortality risk estimates will be of practical use to public health authorities in vulnerability and adaptation planning.

What are the key implications for public health interventions, practice, or policy?

- We find a net increase in temperature-related excess mortality is expected in Canada, that temperature-related mortality is highest under a higher emission scenario, that higher population growth will amplify temperature-related mortality, and that the highest net increase is expected among people over 65.
- These findings should be informative in continued efforts in developing public health policies, and strategies for climate change mitigation and adaptation at both national and local levels.

## Supplementary Information

Below is the link to the electronic supplementary material.

Supplementary file1 (DOCX 135 kb)

## Data Availability

All data used in our study were obtained under a data-sharing agreement between Health Canada and Statistics Canada, and cannot be made publicly available.

## Code availability

R codes used for this study can be made available upon request.

## References

[CR1] Arbuthnott, K., Hajat, S., Heaviside, C., & Vardoulakis, S. (2016). Changes in population susceptibility to heat and cold over time: Assessing adaptation to climate change. *Environmental Health: A Global Access Science Source, 15 Suppl 1*, 33–016–0102–7. 10.1186/s12940-016-0102-7 10.1186/s12940-016-0102-7 PMC4895245 26961541

[CR2] Austin SE, Ford JD, Berrang-Ford L, Biesbroek R, Ross NA (2019). Enabling local public health adaptation to climate change. Social Science & Medicine.

[CR3] Benmarhnia T, Deguen S, Kaufman JS, Smargiassi A (2015). Review article: Vulnerability to heat-related mortality: A systematic review, meta-analysis, and meta-regression analysis. Epidemiology (Cambridge, Mass.).

[CR4] Bush, E., & Lemmen, D. S. (2019). Canada’s changing climate report. Ottawa, ON: Government of Canada. https://changingclimate.ca/CCCR2019/

[CR5] Cakmak, S., Hebbern, C., Pinault, L., Lavigne, E., Vanos, J., Crouse, D. L., & Tjepkema, M. (2018). Associations between long-term PM2.5 and ozone exposure and mortality in the Canadian Census Health and Environment Cohort (CANCHEC), by spatial synoptic classification zone. *Environment International, 111*, 200-211. doi:S0160-4120(17)30895-4 10.1016/j.envint.2017.11.030 29227849

[CR6] Chen K, Vicedo-Cabrera AM, Dubrow R (2020). Projections of ambient temperature- and air pollution-related mortality burden under combined climate change and population aging scenarios: A review. Current Environmental Health Reports.

[CR7] Chen, K., Zhou, L., Chen, X., Ma, Z., Liu, Y., Huang, L., . . . Kinney, P. L. (2016). Urbanization level and vulnerability to heat-related mortality in Jiangsu Province, China. *Environmental Health Perspectives, 124*(12), 1863–1869. EHP204 10.1289/EHP204 PMC5132638 27152420

[CR8] Ebi, K. L., Capon, A., Berry, P., Broderick, C., de Dear, R., Havenith, G., . . . Jay, O. (2021). Hot weather and heat extremes: Health risks. *Lancet (London, England), 398*(10301), 698–708. S0140–6736(21)01208–3 10.1016/S0140-6736(21)01208-3 34419205

[CR9] Gasparrini, A., & Armstrong, B. (2013). Reducing and meta-analysing estimates from distributed lag non-linear models. *BMC Medical Research Methodology, 13*, 1–2288–13–1. 10.1186/1471-2288-13-1 10.1186/1471-2288-13-1 PMC3599933 23297754

[CR10] Gasparrini, A., Guo, Y., Hashizume, M., Kinney, P. L., Petkova, E. P., Lavigne, E., . . . Armstrong, B. G. (2015). Temporal variation in heat-mortality associations: A multicountry study. *Environmental Health Perspectives,*10.1289/ehp.1409070 10.1289/ehp.1409070 PMC4629745 25933359

[CR11] Gasparrini, A., Guo, Y., Sera, F., Vicedo-Cabrera, A. M., Huber, V., Tong, S., . . . Armstrong, B. (2017). Projections of temperature-related excess mortality under climate change scenarios. *The Lancet.Planetary Health, 1*(9), e360-e367. 10.1016/S2542-5196(17)30156-0 10.1016/S2542-5196(17)30156-0 PMC5729020 29276803

[CR12] Gidden, M. J., Riahi, K., Smith, S. J., Fujimori, S., Luderer, G., Kriegler, E., . . . Takahashi, K. (2019). Global emissions pathways under different socioeconomic scenarios for use in CMIP6: A dataset of harmonized emissions trajectories through the end of the century. *Geoscientific Model Development, 12*(4), 1443-1475. 10.5194/gmd-12-1443-2019

[CR13] Guo, Y., Gasparrini, A., Armstrong, B., Li, S., Tawatsupa, B., Tobias, A., . . . Williams, G. (2014). Global variation in the effects of ambient temperature on mortality: A systematic evaluation. *Epidemiology, 25*(6), 781–789. 10.1097/EDE.0000000000000165 10.1097/EDE.0000000000000165 PMC4180721 25166878

[CR14] Guo, Y., Gasparrini, A., Li, S., Sera, F., Vicedo-Cabrera, A., de Sousa Zanotti Stagliorio Coelho, M., . . . Tong, S. (2018). Quantifying excess deaths related to heatwaves under climate change scenarios: A multicountry time series modelling study. *PLoS Medicine, 15*(7) 10.1371/journal.pmed.1002629 10.1371/journal.pmed.1002629 PMC6067704 30063714

[CR15] Harper, S. L., Cunsolo, A., Babujee, A., Coggins, S., De Jongh, E., Rusnak, T., . . . Domínguez Aguilar, M. (2021). Trends and gaps in climate change and health research in North America. *Environmental Research, 199*, 111205. S0013–9351(21)00499–0 10.1016/j.envres.2021.111205 33961824

[CR16] Hempel S, Frieler K, Warszawski L, Schewe J, Piontek F (2013). A trend-preserving bias correction – The ISI-MIP approach. Earth System Dynamics.

[CR17] Hu, K., Guo, Y., Hochrainer-Stigler, S., Liu, W., See, L., Yang, X., . . . Qi, J. (2019). Evidence for urban-rural disparity in temperature-mortality relationships in Zhejiang Province, China. *Environmental Health Perspectives, 127*(3), 37001. 10.1289/EHP3556 10.1289/EHP3556 PMC6768324 30822387

[CR18] KC S, Lutz W (2017). The human core of the Shared Socioeconomic Pathways: Population scenarios by age, sex and level of education for all countries to 2100. Global Environmental Change.

[CR19] Lee, J. Y., Kim, H., Gasparrini, A., Armstrong, B., Bell, M. L., Sera, F., . . . Guo, Y. (2019). Predicted temperature-increase-induced global health burden and its regional variability. *Environment International, 131*10.1016/j.envint.2019.105027 10.1016/j.envint.2019.105027 31351381

[CR20] Marinucci GD, Luber G, Uejio CK, Saha S, Hess JJ (2014). Building resilience against climate effects—A novel framework to facilitate climate readiness in public health agencies. International Journal of Environmental Research and Public Health.

[CR21] Martin SL, Cakmak S, Hebbern CA, Avramescu ML, Tremblay N (2012). Climate change and future temperature-related mortality in 15 Canadian cities. International Journal of Biometeorology.

[CR22] Martínez-Solanas, È., Quijal-Zamorano, M., Achebak, H., Petrova, D., Robine, J. M., Herrmann, F. R., . . . Ballester, J. (2021). Projections of temperature-attributable mortality in Europe: A time series analysis of 147 contiguous regions in 16 countries. *The Lancet.Planetary Health, 5*(7), e446-e454. S2542–5196(21)00150–9 10.1016/S2542-5196(21)00150-9 34245715

[CR23] Onozuka, D., Gasparrini, A., Sera, F., Hashizume, M., & Honda, Y. (2019). Future projections of temperature-related excess out-of-hospital cardiac arrest under climate change scenarios in Japan. *The Science of the Total Environment, 682*, 333-339. S0048-9697(19)32235-1 10.1016/j.scitotenv.2019.05.196 31125746

[CR24] Sera F, Armstrong B, Blangiardo M, Gasparrini A (2019). An extended mixed-effects framework for meta-analysis. Statistics in Medicine.

[CR25] Sera, F., Hashizume, M., Honda, Y., Lavigne, E., Schwartz, J., Zanobetti, A., . . . Gasparrini, A. (2020). Air conditioning and heat-related mortality: A multi-country longitudinal study. *Epidemiology,* 779–787. 10.1097/EDE.0000000000001241 10.1097/EDE.0000000000001241 33003149

[CR26] Statistics Canada (2023). Canadian Community Health Survey - Annual component (CCHS). Retrieved from https://www23.statcan.gc.ca/imdb/p2SV.pl?Function=getSurvey&SDDS=3226. Accessed 10-05-2023

[CR27] Stieb, D. M., Yao, J., Henderson, S. B., Pinault, L., Smith-Doiron, M. H., Robichaud, A., . . . Brook, J. R. (2019). Variability in ambient ozone and fine particle concentrations and population susceptibility among Canadian health regions. *Canadian Journal of Public Health = Revue Canadienne De Sante Publique, 110*(2), 149–158. 10.17269/s41997-018-0169-8 10.17269/s41997-018-0169-8 PMC6964403 30617991

[CR28] Vicedo-Cabrera AM, Sera F, Gasparrini A (2019). Hands-on tutorial on a modeling framework for projections of climate change impacts on health. Epidemiology (Cambridge, Mass.).

[CR29] Wang, P., Tong, H. W., Lee, T. C., & Goggins, W. B. (2022). Projecting future temperature-related mortality using annual time series data: An example from Hong Kong. *Environmental Research, 212*(Pt C), 113351. S0013–9351(22)00678–8 10.1016/j.envres.2022.113351 35490827

[CR30] Watts, N., Amann, M., Ayeb-Karlsson, S., Belesova, K., Bouley, T., Boykoff, M., . . . Costello, A. (2018). The lancet countdown on health and climate change: From 25 years of inaction to a global transformation for public health. *Lancet (London, England), 391*(10120), 581–630. S0140–6736(17)32464–9 10.1016/S0140-6736(17)32464-9 29096948

[CR31] Weinberger KR, Kirwa K, Eliot MN, Gold J, Suh HH, Wellenius GA (2018). Projected changes in temperature-related morbidity and mortality in southern New England. Epidemiology (Cambridge, Mass.).

[CR32] Xing, Q., Sun, Z., Tao, Y., Zhang, X., Miao, S., Zheng, C., & Tong, S. (2020). Impacts of urbanization on the temperature-cardiovascular mortality relationship in Beijing, China. *Environmental Research, 191*, 110234. S0013–9351(20)31131–2 10.1016/j.envres.2020.110234 32956657

[CR33] Xing, Q., Sun, Z., Tao, Y., Shang, J., Miao, S., Xiao, C., & Zheng, C. (2022). Projections of future temperature-related cardiovascular mortality under climate change, urbanization and population aging in Beijing, China. *Environment International, 163*, 107231. S0160–4120(22)00157-X 10.1016/j.envint.2022.107231 35436720

[CR34] Zhao, S., Yu, Y., Lin, P., Liu, H., He, B., Bao, Q., . . . Wang, X. (2021). Datasets for the CMIP6 Scenario Model Intercomparison Project (ScenarioMIP) simulations with the coupled model CAS FGOALS-f3-L. *Advances in Atmospheric Sciences, 38*(2), 329–339. 10.1007/s00376-020-0112-9

