# Supplementary material for: Future temperature-related excess mortality under climate change and population aging scenarios in Canada
Source: Can J Public Health. 2023 Jun 12;114(5):726–36. doi: 10.17269/s41997-023-00782-5 (PMC10484859; doi:10.17269/s41997-023-00782-5)
Supplement: Supplementary file 1 — Supplementary file1 (DOCX 135 kb) [file 41997_2023_782_MOESM1_ESM.docx]

Supplementary Material

**Future temperature-related excess mortality under climate change and population aging scenarios in Canada**

**Authors and affiliations: Christopher Hebbern, Pierre Gosselin, Kai Chen, Hong Chen, Sabit Cakmak, Melissa MacDonald, Jonathan Chagnon, Patrice Dion, Laurent Martel and Eric Lavigne.**

**Table S1**. Population growth scenarios under Shared Socio Economic Pathways (SSPs). A scenario is created by selecting an assumption for each component of population growth. A summary of the assumptions underlying each of the projection scenarios is provided in the table below. From: Statistics Canada. 2019. Population Projections for Canada (2018 to 2100): Technical Report on Methodology and Assumptions, catalogue no. 91-620-X.

|  | Fertility | Mortality | Immigration | Emigration and returning emigration | Temporary emigration | Non-permanent residents |
| --- | --- | --- | --- | --- | --- | --- |
| SSP1 | Medium | Low | Medium | Medium | Unique | Medium |
| SSP2 | Medium | Medium | Medium | Medium | Unique | Medium |
| SSP5 | High | Low | High | Low | Unique | High |

**Table S2.** Yearly average number of deaths by health region in Canada (2000 – 2015).

| Province/Territory & Health region | **Yearly average** |
| --- | --- |
| ***Newfoundland and Labrador*** |  |
| Eastern Regional Health Authority | 2162.1 |
| Central Regional Health Authority | 849.9 |
| Western Regional Health Authority | 672.7 |
| Labrador-Grenfell Regional Health Authority | 181.7 |
| ***Prince Edward Island*** |  |
| Prince Edward Island | 1127.7 |
| ***Nova Scotia*** |  |
| Zone 1 - Western | 1837.6 |
| Zone 2 - Northern | 1372.2 |
| Zone 3 - Eastern | 1650 |
| Zone 4 - Central | 2564.8 |
| ***New Brunswick*** |  |
| Zone 1 (Moncton area) | 1477.3 |
| Zone 2 (Saint John area) | 1459.8 |
| Zone 3 (Fredericton area) | 1304.3 |
| Zone 4 (Edmundston area) | 405.1 |
| Zone 5 (Campbellton area) | 274.1 |
| Zone 6 (Bathurst area) | 598.9 |
| Zone 7 (Miramichi area) | 386.6 |
| ***Québec*** |  |
| Région du Bas-Saint-Laurent | 1791.3 |
| Région du Saguenay - Lac-Saint-Jean | 2130.0 |
| Région de la Capitale-Nationale | 5283.6 |
| Région de la Mauricie et du Centre-du-Québec | 4324.2 |
| Region de l'Estrie | 3478.8 |
| Region de Montréal | 15090.7 |
| Region de l'Outaouais | 2321.9 |
| Région de l'Abitibi-Témiscamingue | 1077 |
| Région de la Cote-Nord | 613.8 |
| Région du Nord-du-Québec | 64.6 |
| Région de la Gaspésie - Iles-de-la-Madeleine | 970.8 |
| Région de la Chaudière-Appalaches | 2852.4 |
| Région de Laval | 2487 |
| Région de Lanaudière | 2831.3 |
| Région des Laurentides | 3477.4 |
| Région de la Montérégie | 8252.4 |
| Région du Nunavik | 43.1 |
| Région des Terres-Cries-de-la-Baie-James | 54 |
| ***Ontario*** |  |
| The District of Algoma Health Unit | 1044.6 |
| Brant County Health Unit | 1009.9 |
| Durham Regional Health Unit | 2903.7 |
| Grey Bruce Health Unit | 1322.6 |
| Haldimand-Norfolk Health Unit | 839 |
| Haliburton, Kawartha, Pine Ridge District Health Unit | 1532.2 |
| Halton Regional Health Unit | 2342.1 |
| City of Hamilton Health Unit | 3423.9 |
| Hastings and Prince Edward Counties Health Unit | 1461.2 |
| Huron County Health Unit | 541 |
| Chatham-Kent Health Unit | 898.5 |
| Kingston, Frontenac and Lennox and Addington Health Unit | 1424.6 |
| Lambton Health Unit | 1045.8 |
| Leeds, Grenville and Lanark District Health Unit | 1400.8 |
| Middlesex-London Health Unit | 2718.4 |
| Niagara Regional Area Health Unit | 3636.1 |
| North Bay Parry Sound District Health Unit | 1024.5 |
| Northwestern Health Unit | 565.6 |
| City of Ottawa Health Unit | 4150.4 |
| Peel Regional Health Unit | 4164.6 |
| Perth District Health Unit | 574.8 |
| Peterborough County Health Unit | 1102.8 |
| Porcupine Health Unit | 641.4 |
| Renfrew County and District Health Unit | 776.7 |
| The Eastern Ontario Health Unit | 1502 |
| Simcoe Muskoka District Health Unit | 3359.9 |
| Sudbury and District Health Unit | 1490.8 |
| Thunder Bay District Health Unit | 1193.6 |
| Timiskaming Health Unit | 301.9 |
| Waterloo Health Unit | 2644.4 |
| Wellington-Dufferin-Guelph Health Unit | 1566.4 |
| Windsor-Essex County Health Unit | 2705.1 |
| York Regional Health Unit | 3466.6 |
| Oxford Elgin St. Thomas Health Unit | 1438.9 |
| City of Toronto Health Unit | 14062 |
| ***Manitoba*** |  |
| Winnipeg Regional Health Authority | 5258.3 |
| Prairie Mountain Health | 1623.3 |
| Interlake-Eastern Regional Health Authority | 923.9 |
| Northern Regional Health Authority | 335.2 |
| Southern Health | 1101 |
| ***Saskatchewan*** |  |
| Sun Country Regional Health Authority | 517.9 |
| Five Hills Regional Health Authority | 561.1 |
| Cypress Regional Health Authority | 411.3 |
| Regina Qu'Appelle Regional Health Authority | 1861.3 |
| Sunrise Regional Health Authority | 719.6 |
| Saskatoon Regional Health Authority | 2072.6 |
| Heartland Regional Health Authority | 430.4 |
| Kelsey Trail Regional Health Authority | 422.3 |
| Prince Albert Parkland Regional Health Authority | 557 |
| Prairie North Regional Health Authority | 471.3 |
| Mamawetan Churchill River Regional Health Authority | 70.2 |
| Keewatin Yatth Regional Health Authority | 47.6 |
| Athabasca Health Authority | 7.8 |
| ***Alberta*** |  |
| South Zone | 1837.8 |
| Calgary Zone | 5530.1 |
| Central Zone | 2714.5 |
| Edmonton Zone | 5558.8 |
| North Zone | 1874.2 |
| ***British Columbia*** |  |
| East Kootenay Health Service Delivery Area | 594.8 |
| Kootenay-Boundary Health Service Delivery Area | 695.5 |
| Okanagan Health Service Delivery Area | 3080.3 |
| Thompson/Cariboo Health Service Delivery Area | 1647.9 |
| Fraser East Health Service Delivery Area | 1916.2 |
| Fraser North Health Service Delivery Area | 3203.8 |
| Fraser South Health Service Delivery Area | 3858.3 |
| Richmond Health Service Delivery Area | 846.1 |
| Vancouver Health Service Delivery Area | 3513.1 |
| North Shore/Coast Garibaldi Health Service Delivery Area | 1815.8 |
| South Vancouver Island Health Service Delivery Area | 3069.9 |
| Central Vancouver Island Health Service Delivery Area | 2282.2 |
| North Vancouver Island Health Service Delivery Area | 853.6 |
| Northwest Health Service Delivery Area | 417 |
| Northern Interior Health Service Delivery Area | 828.7 |
| Northeast Health Service Delivery Area | 318.2 |
| ***Yukon*** |  |
| Yukon | 151.3 |
| ***Northwest Territories*** |  |
| Northwest Territories | 127.9 |
| ***Nunavut*** |  |
| Nunavut | 83.2 |

**Table S3.** Current mean temperature and projected increase (°C) by SSP and health region in Canada.

| Province/Territory & Health region | Current  temperature  (2010–19) | **SSP2-4.5** | | **SSP5-8.5** | |
| --- | --- | --- | --- | --- | --- |
|  |  | 2050-59 | 2090-99 | 2050-59 | 2090-99 |
| ***Newfoundland and Labrador*** |  |  |  |  |  |
| Eastern Regional Health Authority | 6.0 | 1.3 | 2.0 | 1.9 | 4.5 |
| Central Regional Health Authority | 5.1 | 1.5 | 2.3 | 2.2 | 5.0 |
| Western Regional Health Authority | 3.8 | 1.8 | 2.5 | 2.4 | 5.3 |
| Labrador-Grenfell Regional Health Authority | -2.2 | 1.8 | 3.0 | 2.7 | 6.2 |
| ***Prince Edward Island*** |  |  |  |  |  |
| Prince Edward Island | 6.7 | 1.8 | 2.6 | 2.3 | 5.3 |
| ***Nova Scotia*** |  |  |  |  |  |
| Zone 1 - Western | 8.3 | 1.7 | 2.4 | 2.1 | 4.8 |
| Zone 2 - Northern | 6.3 | 1.8 | 2.5 | 2.2 | 5.1 |
| Zone 3 - Eastern | 6.8 | 1.9 | 2.5 | 2.3 | 5.2 |
| Zone 4 - Central | 6.9 | 1.8 | 2.5 | 2.2 | 5.0 |
| ***New Brunswick*** |  |  |  |  |  |
| Zone 1 (Moncton area) | 6.1 | 1.8 | 2.6 | 2.3 | 5.4 |
| Zone 2 (Saint John area) | 5.9 | 1.7 | 2.5 | 2.2 | 5.3 |
| Zone 3 (Fredericton area) | 5.7 | 1.7 | 2.6 | 2.3 | 5.4 |
| Zone 4 (Edmundston area) | 4.2 | 1.7 | 2.8 | 2.5 | 5.8 |
| Zone 5 (Campbellton area) | 3.4 | 1.7 | 2.8 | 2.5 | 5.8 |
| Zone 6 (Bathurst area) | 4.8 | 1.8 | 2.7 | 2.5 | 5.7 |
| Zone 7 (Miramichi area) | 5.0 | 1.8 | 2.7 | 2.4 | 5.6 |
| ***Québec*** |  |  |  |  |  |
| Région du Bas-Saint-Laurent | 3.6 | 1.7 | 2.8 | 2.5 | 6.0 |
| Région du Saguenay - Lac-Saint-Jean | 2.9 | 1.7 | 2.9 | 2.5 | 6.1 |
| Région de la Capitale-Nationale | 4.5 | 1.7 | 2.8 | 2.5 | 5.9 |
| Région de la Mauricie et du Centre-du-Québec | 5.2 | 1.7 | 2.8 | 2.4 | 5.8 |
| Region de l'Estrie | 5.9 | 1.7 | 2.7 | 2.4 | 5.6 |
| Region de Montréal | 6.9 | 1.7 | 2.7 | 2.4 | 5.7 |
| Region de l'Outaouais | 6.8 | 1.7 | 2.7 | 2.4 | 5.7 |
| Région de l'Abitibi-Témiscamingue | 2.3 | 1.6 | 2.8 | 2.4 | 6.1 |
| Région de la Cote-Nord | 1.8 | 1.8 | 2.9 | 2.6 | 6.0 |
| Région du Nord-du-Québec | 0.7 | 1.7 | 3.0 | 2.5 | 6.2 |
| Région de la Gaspésie - Iles-de-la-Madeleine | 4.4 | 1.8 | 2.7 | 2.5 | 5.6 |
| Région de la Chaudière-Appalaches | 4.5 | 1.7 | 2.8 | 2.5 | 5.9 |
| Région de Laval | 6.3 | 1.7 | 2.8 | 2.4 | 5.7 |
| Région de Lanaudière | 6.4 | 1.7 | 2.8 | 2.4 | 5.7 |
| Région des Laurentides | 6.3 | 1.7 | 2.8 | 2.4 | 5.7 |
| Région de la Montérégie | 6.3 | 1.7 | 2.8 | 2.4 | 5.7 |
| Région du Nunavik | -4.0 | 1.9 | 3.3 | 3.0 | 7.1 |
| Région des Terres-Cries-de-la-Baie-James | 0.3 | 1.7 | 3.0 | 2.5 | 6.2 |
| ***Ontario*** |  |  |  |  |  |
| The District of Algoma Health Unit | 4.5 | 1.6 | 2.8 | 2.5 | 5.9 |
| Brant County Health Unit | 8.3 | 1.7 | 2.5 | 2.3 | 5.3 |
| Durham Regional Health Unit | 8.9 | 1.7 | 2.6 | 2.3 | 5.4 |
| Grey Bruce Health Unit | 7.4 | 1.7 | 2.6 | 2.3 | 5.5 |
| Haldimand-Norfolk Health Unit | 9.5 | 1.7 | 2.6 | 2.3 | 5.3 |
| Haliburton, Kawartha, Pine Ridge District Health Unit | 8.7 | 1.7 | 2.6 | 2.3 | 5.4 |
| Halton Regional Health Unit | 9.5 | 1.7 | 2.6 | 2.3 | 5.3 |
| City of Hamilton Health Unit | 9.5 | 1.7 | 2.6 | 2.3 | 5.3 |
| Hastings and Prince Edward Counties Health Unit | 8.0 | 1.7 | 2.6 | 2.4 | 5.5 |
| Huron County Health Unit | 8.0 | 1.6 | 2.5 | 2.2 | 5.3 |
| Chatham-Kent Health Unit | 9.9 | 1.6 | 2.4 | 2.2 | 5.2 |
| Kingston, Frontenac and Lennox and Addington Health Unit | 8.1 | 1.7 | 2.6 | 2.4 | 5.5 |
| Lambton Health Unit | 9.5 | 1.6 | 2.5 | 2.2 | 5.2 |
| Leeds, Grenville and Lanark District Health Unit | 7.8 | 1.7 | 2.7 | 2.4 | 5.6 |
| Middlesex-London Health Unit | 8.6 | 1.6 | 2.5 | 2.2 | 5.2 |
| Niagara Regional Area Health Unit | 9.8 | 1.7 | 2.6 | 2.3 | 5.3 |
| North Bay Parry Sound District Health Unit | 5.2 | 1.6 | 2.7 | 2.4 | 5.9 |
| Northwestern Health Unit | 3.3 | 1.6 | 2.8 | 2.6 | 6.2 |
| City of Ottawa Health Unit | 6.8 | 1.7 | 2.7 | 2.4 | 5.7 |
| Peel Regional Health Unit | 7.8 | 1.7 | 2.6 | 2.3 | 5.4 |
| Perth District Health Unit | 7.9 | 1.7 | 2.5 | 2.2 | 5.3 |
| Peterborough County Health Unit | 7.3 | 1.7 | 2.6 | 2.3 | 5.5 |
| Porcupine Health Unit | 2.7 | 1.6 | 2.9 | 2.4 | 6.2 |
| Renfrew County and District Health Unit | 5.8 | 1.7 | 2.7 | 2.4 | 5.8 |
| The Eastern Ontario Health Unit | 6.8 | 1.7 | 2.7 | 2.4 | 5.7 |
| Simcoe Muskoka District Health Unit | 7.2 | 1.7 | 2.6 | 2.3 | 5.5 |
| Sudbury and District Health Unit | 5.8 | 1.6 | 2.7 | 2.4 | 5.9 |
| Thunder Bay District Health Unit | 3.4 | 1.6 | 2.9 | 2.5 | 6.1 |
| Timiskaming Health Unit | 2.4 | 1.6 | 2.9 | 2.4 | 6.1 |
| Waterloo Health Unit | 8.3 | 1.7 | 2.5 | 2.3 | 5.3 |
| Wellington-Dufferin-Guelph Health Unit | 7.0 | 1.7 | 2.6 | 2.3 | 5.4 |
| Windsor-Essex County Health Unit | 9.9 | 1.6 | 2.4 | 2.2 | 5.2 |
| York Regional Health Unit | 8.9 | 1.7 | 2.6 | 2.3 | 5.4 |
| Oxford Elgin St. Thomas Health Unit | 8.6 | 1.6 | 2.5 | 2.2 | 5.2 |
| City of Toronto Health Unit | 8.9 | 1.7 | 2.6 | 2.3 | 5.4 |
| ***Manitoba*** |  |  |  |  |  |
| Winnipeg Regional Health Authority | 3.4 | 1.7 | 3.0 | 2.7 | 6.3 |
| Prairie Mountain Health | 3.7 | 1.7 | 2.9 | 2.8 | 6.3 |
| Interlake-Eastern Regional Health Authority | 3.1 | 1.7 | 2.9 | 2.7 | 6.3 |
| Northern Regional Health Authority | 0.9 | 1.6 | 2.8 | 2.7 | 6.5 |
| Southern Health | 4.1 | 1.7 | 3.0 | 2.8 | 6.3 |
| ***Saskatchewan*** |  |  |  |  |  |
| Sun Country Regional Health Authority | 4.6 | 1.7 | 2.9 | 2.7 | 6.0 |
| Five Hills Regional Health Authority | 4.3 | 1.7 | 2.8 | 2.7 | 5.9 |
| Cypress Regional Health Authority | 4.6 | 1.8 | 2.9 | 2.7 | 5.8 |
| Regina Qu'Appelle Regional Health Authority | 4.5 | 1.7 | 2.8 | 2.7 | 6.0 |
| Sunrise Regional Health Authority | 2.2 | 1.6 | 2.8 | 2.7 | 6.3 |
| Saskatoon Regional Health Authority | 3.0 | 1.7 | 2.8 | 2.6 | 6.1 |
| Heartland Regional Health Authority | 4.0 | 1.8 | 2.8 | 2.7 | 5.9 |
| Kelsey Trail Regional Health Authority | 2.1 | 1.6 | 2.7 | 2.6 | 6.2 |
| Prince Albert Parkland Regional Health Authority | 1.6 | 1.6 | 2.7 | 2.6 | 6.2 |
| Prairie North Regional Health Authority | 2.5 | 1.7 | 2.8 | 2.6 | 6.0 |
| Mamawetan Churchill River Regional Health Authority | 0.1 | 1.6 | 2.7 | 2.7 | 6.4 |
| Keewatin Yatth Regional Health Authority | 1.1 | 1.6 | 2.6 | 2.6 | 6.2 |
| Athabasca Health Authority | -3.0 | 1.5 | 2.7 | 2.6 | 6.7 |
| ***Alberta*** |  |  |  |  |  |
| South Zone | 5.7 | 1.9 | 2.9 | 2.6 | 5.6 |
| Calgary Zone | 4.0 | 1.7 | 2.6 | 2.5 | 5.5 |
| Central Zone | 3.6 | 1.8 | 2.7 | 2.5 | 5.6 |
| Edmonton Zone | 3.9 | 1.8 | 2.7 | 2.5 | 5.7 |
| North Zone | 1.2 | 1.7 | 2.7 | 2.7 | 6.2 |
| ***British Columbia*** |  |  |  |  |  |
| East Kootenay Health Service Delivery Area | 4.4 | 1.6 | 2.4 | 2.5 | 5.4 |
| Kootenay-Boundary Health Service Delivery Area | 5.0 | 1.6 | 2.4 | 2.5 | 5.3 |
| Okanagan Health Service Delivery Area | 4.2 | 1.6 | 2.3 | 2.4 | 5.2 |
| Thompson/Cariboo Health Service Delivery Area | 6.3 | 1.6 | 2.3 | 2.4 | 5.2 |
| Fraser East Health Service Delivery Area | 8.8 | 1.5 | 2.2 | 2.3 | 5.0 |
| Fraser North Health Service Delivery Area | 10.7 | 1.5 | 2.1 | 2.2 | 4.9 |
| Fraser South Health Service Delivery Area | 10.7 | 1.5 | 2.1 | 2.2 | 4.9 |
| Richmond Health Service Delivery Area | 10.6 | 1.5 | 2.1 | 2.2 | 4.8 |
| Vancouver Health Service Delivery Area | 10.6 | 1.5 | 2.1 | 2.2 | 4.8 |
| North Shore/Coast Garibaldi Health Service Delivery Area | 10.6 | 1.5 | 2.1 | 2.2 | 4.8 |
| South Vancouver Island Health Service Delivery Area | 10.7 | 1.4 | 2.0 | 2.1 | 4.6 |
| Central Vancouver Island Health Service Delivery Area | 10.3 | 1.4 | 2.0 | 2.1 | 4.6 |
| North Vancouver Island Health Service Delivery Area | 10.7 | 1.4 | 2.0 | 2.2 | 4.7 |
| Northwest Health Service Delivery Area | 7.6 | 1.3 | 2.0 | 2.0 | 4.3 |
| Northern Interior Health Service Delivery Area | 5.7 | 1.6 | 2.4 | 2.3 | 5.2 |
| Northeast Health Service Delivery Area | 2.9 | 1.8 | 2.5 | 2.5 | 5.5 |
| ***Yukon*** |  |  |  |  |  |
| Yukon | -1.6 | 1.6 | 2.6 | 2.6 | 5.8 |
| ***Northwest Territories*** |  |  |  |  |  |
| Northwest Territories | -3.5 | 1.8 | 2.8 | 2.9 | 6.8 |
| ***Nunavut*** |  |  |  |  |  |
| Nunavut | -8.1 | 1.8 | 3.5 | 3.0 | 7.7 |

**Table S4.** Heat-related, cold-related, and net change in excess non-accidental mortality (%) with 95% empirical Confidence Interval (eCI) by period with no population change and under two climate change scenarios in Canada.

| Age group | Scenario | Effect | Period | | |
| --- | --- | --- | --- | --- | --- |
|  |  |  | 2010-2019 | 2050-2059 | 2090-2099 |
| All | SSP2-4.5 | Heat | 0.41 (0.23, 0.59) | 1.01 (0.7, 1.31) | 1.48 (1.08, 1.87) |
|  |  | Cold | 3.8 (1.1, 6.51) | 3.57 (1.04, 6.11) | 3.55 (1.02, 6.09) |
|  |  | Net | - | 0.23 (0.01, 0.45) | 0.53 (0.2, 0.87) |
|  | SSP5-8.5 | Heat | 0.41 (0.24, 0.59) | 1.38 (0.98, 1.77) | 3.91 (3.03, 4.79) |
|  |  | Cold | 3.81 (1.08, 6.53) | 3.46 (1.02, 5.91) | 3.25 (1.11, 5.38) |
|  |  | Net | - | 0.46 (0.14, 0.78) | 2.61 (1.65, 3.57) |
| 65 and over | SSP2-4.5 | Heat | 0.75 (0.45, 1.06) | 0.62 (0.42, 0.83) | 0.76 (0.52, 1) |
|  |  | Cold | 4.47 (-0.34, 9.28) | 5.02 (2.23, 7.81) | 4.73 (1.75, 7.72) |
|  |  | Net | - | 0.1 (-0.09, 0.29) | 0.1 (-0.12, 0.32) |
|  | SSP5-8.5 | Heat | 1.12 (0.67, 1.58) | 1.3 (0.85, 1.74) | 1.25 (0.84, 1.66) |
|  |  | Cold | 4.32 (-0.5, 9.13) | 4.71 (1.98, 7.44) | 4.57 (1.74, 7.4) |
|  |  | Net | - | 0.2 (-0.01, 0.42) | 0.28 (0.05, 0.52) |
| Under 65 | SSP2-4.5 | Heat | 0.33 (-0.14, 0.79) | 0.35 (-0.6, 1.29) | 0.45 (-0.72, 1.63) |
|  |  | Cold | -4.56 (-11.45, 2.33) | -4.02 (-10.39, 2.35) | -3.65 (-9.49, 2.2) |
|  |  | Net | - | 0.32 (-0.37, 1) | 0.63 (-0.36, 1.61) |
|  | SSP5-8.5 | Heat | 0.34 (-0.11, 0.78) | 0.24 (-1.06, 1.54) | -1.23 (-5.3, 2.84) |
|  |  | Cold | -4.55 (-11.51, 2.41) | -3.83 (-9.98, 2.32) | -2.85 (-7.65, 1.95) |
|  |  | Net | - | 0.52 (-0.62, 1.67) | 0.01 (-3.73, 3.75) |

**Table S5.** Model fit sensitivity analysis using different degrees of freedom for the lag periods and different temperature-mortality associations

| **Model** | **^AIC^** |
| --- | --- |
|  |  |
| Main model (Knots at the 10^th^, 75^th^ and 90^th^ percentile of temperature distribution df = 3; equally spaced knots for the lag function, df = 3) | 46,754 |
| Alternative model (Knots at the 10^th^, 50^th^ and 90^th^ percentile of temperature distribution df = 3; equally spaced knots for the lag function, df = 3) | 47,050 |
| Alternative model (Knots at the 10^th^, 75^th^ and 90^th^ percentile of temperature distribution df = 4; equally spaced knots for the lag function, df = 3) | 46,805 |
| Alternative model (Knots at the 10^th^, 75^th^ and 90^th^ percentile of temperature distribution df = 5; equally spaced knots for the lag function, df = 3) | 46,860 |

^1^ Akaike Information Criteria

**Table S6.** Net change in excess non-accidental mortality (%) with 95% empirical Confidence Interval (eCI) during 2090-2099 compared to 2010-2019 under two climate change scenarios by health region in Canada.

| Province/Territory & Health region | **SSP2-4.5** | **SSP5-8.5** |
| --- | --- | --- |
| ***Newfoundland and Labrador*** |  |  |
| Eastern Regional Health Authority | -1.29 (-3.36 to 1.03) | -1.77 (-7.49 to 3.45) |
| Central Regional Health Authority | -1.9 (-4.59 to 1.18) | -2.88 (-9.33 to 4.91) |
| Western Regional Health Authority | 0.69 (-4.09 to 5.6) | 4.39 (-9.26 to 16.36) |
| Labrador-Grenfell Regional Health Authority | -1.1 (-16.38 to 7.95) | -1.91 (-38.97 to 25.54) |
| ***Prince Edward Island*** |  |  |
| Prince Edward Island | -2.14 (-6.27 to 1.97) | -3.46 (-15.75 to 4.96) |
| ***Nova Scotia*** |  |  |
| Zone 1 - Western | 2.47 (-0.64 to 6.16) | 7.23 (-1.1 to 14.96) |
| Zone 2 - Northern | -0.92 (-4.65 to 3.13) | -0.97 (-13.05 to 8.72) |
| Zone 3 - Eastern | -1.45 (-4.23 to 2.31) | -4.69 (-14.85 to 3.58) |
| Zone 4 - Central | 1.22 (-1.58 to 5.14) | 2.7 (-6.09 to 10.23) |
| ***New Brunswick*** |  |  |
| Zone 1 (Moncton area) | 2.4 (-1.24 to 6.55) | 6.79 (-2.03 to 14.98) |
| Zone 2 (Saint John area) | -3.04 (-7.46 to -0.02) | -5.79 (-17.4 to 3.01) |
| Zone 3 (Fredericton area) | 0.41 (-4.36 to 5.34) | -1.52 (-15.72 to 6.67) |
| Zone 4 (Edmundston area) | 3.29 (-6.48 to 17.7) | 8.11 (-24.16 to 35.37) |
| Zone 5 (Campbellton area) | 0.8 (-9.52 to 16.15) | 3.02 (-29.89 to 36.2) |
| Zone 6 (Bathurst area) | -0.67 (-7.85 to 6.83) | -3.25 (-24.28 to 9.92) |
| Zone 7 (Miramichi area) | 5.44 (-1.32 to 18.96) | 15.64 (-1.01 to 47.32) |
| ***Québec*** |  |  |
| Région du Bas-Saint-Laurent | 1.6 (-1.4 to 5.1) | 4.05 (-3.51 to 12.1) |
| Région du Saguenay - Lac-Saint-Jean | 0.87 (-1.81 to 3.61) | 3.83 (-2.94 to 10.98) |
| Région de la Capitale-Nationale | 0.62 (-0.82 to 2.55) | 2.66 (-1.6 to 7.68) |
| Région de la Mauricie et du Centre-du-Québec | 0.38 (-2.03 to 2.52) | 3.51 (-1.76 to 8.08) |
| Region de l'Estrie | 0.85 (-0.81 to 3.28) | 2.53 (-2.63 to 7.94) |
| Region de Montreal | 1.32 (-0.12 to 3.66) | 5.55 (1.72 to 10.14) |
| Region de l'Outaouais | 2 (-0.08 to 5.01) | 5.03 (-1.01 to 10.33) |
| Région de l'Abitibi-Témiscamingue | 1.73 (-1.45 to 5.62) | 6.32 (-2.71 to 16.12) |
| Région de la Cote-Nord | -4.95 (-20.16 to 2.05) | -14.5 (-67.57 to 4.51) |
| Région du Nord-du-Québec | 5.11 (-9.87 to 36.78) | 10.96 (-49.14 to 58.63) |
| Région de la Gaspésie - Iles-de-la-Madeleine | -0.62 (-5.66 to 4.72) | 0.17 (-13.61 to 13.39) |
| Région de la Chaudière-Appalaches | 0.96 (-0.87 to 3.2) | 4.02 (-1.24 to 9.45) |
| Région de Laval | 0.68 (-1.9 to 3.6) | 3.87 (-3.27 to 10.08) |
| Région de Lanaudiere | 2.49 (-0.1 to 5.91) | 7.91 (1.54 to 14.72) |
| Région des Laurentides | 1.91 (-0.17 to 5.3) | 6.43 (0.61 to 13.01) |
| Région de la Montérégie | 0.86 (-0.44 to 3.3) | 4.75 (1.1 to 10.04) |
| Région du Nunavik | 21.67 (-30.7 to 440.33) | 29.49 (-233.61 to 831.08) |
| Région des Terres-Cries-de-la-Baie-James | - | - |
| ***Ontario*** |  |  |
| The District of Algoma Health Unit | -1.02 (-5.15 to 1.87) | 2.05 (-6.48 to 7.36) |
| Brant County Health Unit | 4.45 (-1.27 to 12.08) | 10.46 (-2.29 to 21.55) |
| Durham Regional Health Unit | 0.89 (-1.52 to 4.1) | 3.33 (-3.02 to 9.21) |
| Grey Bruce Health Unit | -0.31 (-3.52 to 2.74) | 1.89 (-6.77 to 8.32) |
| Haldimand-Norfolk Health Unit | -0.22 (-5.84 to 5.11) | 0.64 (-13.21 to 9.11) |
| Haliburton, Kawartha, Pine Ridge District Health Unit | 1.65 (-2.14 to 6.57) | 3.41 (-8.16 to 12.55) |
| Halton Regional Health Unit | -0.93 (-4.06 to 2.6) | -2.75 (-12.18 to 3.31) |
| City of Hamilton Health Unit | 1.23 (-0.84 to 4.25) | 3.39 (-3.63 to 9.67) |
| Hastings and Prince Edward Counties Health Unit | -0.45 (-4.46 to 3.74) | -0.72 (-13.09 to 7.37) |
| Huron County Health Unit | -1.67 (-9.24 to 3.62) | -3.48 (-30.35 to 7.69) |
| Chatham-Kent Health Unit | 1.9 (-2.47 to 6.8) | 6.35 (-4.37 to 14.99) |
| Kingston, Frontenac and Lennox and Addington Health Unit | -0.82 (-5.54 to 3.56) | -2.96 (-18.56 to 5.45) |
| Lambton Health Unit | -0.1 (-3.77 to 3.98) | 0.06 (-14.68 to 8.22) |
| Leeds, Grenville and Lanark District Health Unit | 2.49 (-1.92 to 7.75) | 7.09 (-3.52 to 15.41) |
| Middlesex-London Health Unit | 2.13 (-0.28 to 6.29) | 7.22 (1.48 to 14.16) |
| Niagara Regional Area Health Unit | -1.04 (-3.65 to 0.98) | -0.2 (-5.71 to 4.33) |
| North Bay Parry Sound District Health Unit | 6.41 (0.72 to 13.04) | 13.15 (0.09 to 26.56) |
| Northwestern Health Unit | 1.25 (-3.82 to 6.06) | 3.29 (-13.32 to 14.01) |
| City of Ottawa Health Unit | 0.25 (-2.54 to 3.5) | -0.32 (-7.5 to 4.48) |
| Peel Regional Health Unit | 0.14 (-1.87 to 2.43) | 1.6 (-3.24 to 6.07) |
| Perth District Health Unit | -0.62 (-12.84 to 7.5) | 1.57 (-30.76 to 16.96) |
| Peterborough County Health Unit | -4.34 (-10.51 to 0.03) | -12.71 (-35.85 to -2.12) |
| Porcupine Health Unit | 3.09 (-2.49 to 12) | 5.63 (-7.26 to 19.4) |
| Renfrew County and District Health Unit | -3.15 (-7.9 to -0.01) | -5.91 (-23.15 to 2.65) |
| The Eastern Ontario Health Unit | -0.18 (-4.26 to 3.24) | 1.53 (-8.12 to 8.59) |
| Simcoe Muskoka District Health Unit | 1.76 (-0.55 to 5.18) | 4.18 (-2.49 to 10.67) |
| Sudbury and District Health Unit | 1.45 (-1.26 to 4.84) | 6.57 (-1.66 to 14.34) |
| Thunder Bay District Health Unit | 1.24 (-3.04 to 4.76) | 1.36 (-9.8 to 8.54) |
| Timiskaming Health Unit | 4.93 (-2.83 to 20.68) | 10.9 (-16.07 to 39.52) |
| Waterloo Health Unit | 2.12 (-0.46 to 6.07) | 6.32 (0.2 to 13.27) |
| Wellington-Dufferin-Guelph Health Unit | 0.96 (-1.92 to 4.18) | 3.66 (-4.79 to 9.76) |
| Windsor-Essex County Health Unit | 0.3 (-2.2 to 2.92) | 3.47 (-3.27 to 8.88) |
| York Regional Health Unit | 0.84 (-1.24 to 4.05) | 1.94 (-3.98 to 7.7) |
| Oxford Elgin St. Thomas Health Unit | -4.47 (-16.59 to 0.93) | -10.52 (-59.87 to 0.91) |
| City of Toronto Health Unit | 1.2 (-0.2 to 2.97) | 3.58 (0.17 to 6.68) |
| ***Manitoba*** |  |  |
| Winnipeg Regional Health Authority | 1 (-0.66 to 3.54) | 3.53 (-0.15 to 7.58) |
| Prairie Mountain Health | -0.66 (-3.72 to 1.78) | -1.15 (-12.07 to 5.12) |
| Interlake-Eastern Regional Health Authority | -1.12 (-5.37 to 2.57) | -1.26 (-13.63 to 6.08) |
| Northern Regional Health Authority | 0.04 (-4.12 to 4.14) | 1.96 (-11.96 to 11.08) |
| Southern Health | 2.83 (-1.25 to 7.54) | 8.22 (0.28 to 18.28) |
| ***Saskatchewan*** |  |  |
| Sun Country Regional Health Authority | -1.57 (-6.72 to 1.86) | -1.47 (-17.32 to 5.99) |
| Five Hills Regional Health Authority | -1.24 (-7.02 to 3.49) | -1.64 (-17.81 to 8.46) |
| Cypress Regional Health Authority | -0.07 (-4.75 to 4.01) | 1.69 (-12.31 to 11.11) |
| Regina Qu'Appelle Regional Health Authority | -0.47 (-3.34 to 2.34) | 0.34 (-6.92 to 5.78) |
| Sunrise Regional Health Authority | -0.49 (-3.73 to 2.82) | 1.76 (-4.22 to 8.52) |
| Saskatoon Regional Health Authority | -0.76 (-3.31 to 1.62) | -1.03 (-9.46 to 4.28) |
| Heartland Regional Health Authority | 3.86 (-2.35 to 12.48) | 9.39 (-3.74 to 24.17) |
| Kelsey Trail Regional Health Authority | 0.91 (-3.33 to 5.32) | 4.91 (-2.56 to 14.57) |
| Prince Albert Parkland Regional Health Authority | -1.77 (-7.41 to 2.78) | -5.69 (-34.68 to 5.26) |
| Prairie North Regional Health Authority | -0.21 (-6.34 to 6.07) | 1.94 (-15.34 to 14.39) |
| Mamawetan Churchill River Regional Health Authority | -5.61 (-491.16 to 50.17) | -91.75 (-4073.24 to 74.21) |
| Keewatin Yatth Regional Health Authority | 7.8 (-78.28 to 74.37) | 5.03 (-1731.47 to 113.73) |
| Athabasca Health Authority | - | - |
| ***Alberta*** |  |  |
| South Zone | 0.9 (-1.75 to 3.42) | 2.27 (-5.7 to 8.49) |
| Calgary Zone | -1.04 (-2.84 to 0.44) | -1.9 (-6.77 to 2.48) |
| Central Zone | 0.83 (-0.95 to 3.69) | 4.38 (-0.52 to 9.83) |
| Edmonton Zone | 0.87 (-0.54 to 3.03) | 3.23 (-0.23 to 7.29) |
| North Zone | -1.87 (-5.32 to 0.79) | -4.69 (-15.89 to 2.66) |
| ***British Columbia*** |  |  |
| East Kootenay Health Service Delivery Area | -1.57 (-5.47 to 2.13) | -1.37 (-11.98 to 7.93) |
| Kootenay-Boundary Health Service Delivery Area | 2.24 (-1.06 to 7.35) | 8.02 (-0.36 to 20.71) |
| Okanagan Health Service Delivery Area | -0.38 (-2.38 to 1.67) | 0.08 (-5.95 to 6.02) |
| Thompson/Cariboo Health Service Delivery Area | 0.73 (-2.22 to 3.62) | 2.91 (-5.07 to 8.24) |
| Fraser East Health Service Delivery Area | 0.72 (-1.35 to 3.96) | 5.4 (-2.17 to 13.42) |
| Fraser North Health Service Delivery Area | -0.33 (-3.02 to 2.13) | 0.63 (-5.72 to 6.71) |
| Fraser South Health Service Delivery Area | 0.85 (-1.79 to 3.7) | 4.33 (-2.13 to 11.67) |
| Richmond Health Service Delivery Area | -0.07 (-5.38 to 7.1) | 0.94 (-20.71 to 18.04) |
| Vancouver Health Service Delivery Area | -0.09 (-2.62 to 2.31) | 3.38 (-2.92 to 10.75) |
| North Shore/Coast Garibaldi Health Service Delivery Area | 1.9 (-0.87 to 6.45) | 6.47 (-3.19 to 16.18) |
| South Vancouver Island Health Service Delivery Area | -1.69 (-5.15 to 1.14) | -2.95 (-12.15 to 4.39) |
| Central Vancouver Island Health Service Delivery Area | -0.47 (-3.51 to 2.77) | 0.06 (-6.84 to 6.82) |
| North Vancouver Island Health Service Delivery Area | 1.88 (-3.1 to 8.05) | 7.37 (-12.23 to 22.34) |
| Northwest Health Service Delivery Area | 3.28 (-3.07 to 12.51) | 9.03 (-7.03 to 28.46) |
| Northern Interior Health Service Delivery Area | 3.09 (-0.51 to 8.01) | 8.65 (0.01 to 18.68) |
| Northeast Health Service Delivery Area | -3.06 (-13.28 to 1.68) | -7.43 (-47.55 to 7.21) |
| ***Yukon*** |  |  |
| Yukon | -0.41 (-14.52 to 6.75) | 1.03 (-58.92 to 20.45) |
| ***Northwest Territories*** |  |  |
| Northwest Territories | 2.03 (-9.61 to 23.53) | 4.66 (-22.67 to 51.05) |
| ***Nunavut*** |  |  |
| Nunavut | - | - |

**Table S7.** Percentage change in net difference in excess mortality (%) in 2090-99 compared to 2010-19 under SSP5-8.5 per interquartile range (IQR) increase of the county-level characteristics and the significance test of effect modification, with 95% empirical Confidence Interval (eCI).

| County-level characteristics | IQR | Non-accidental mortality | | Cardiovascular mortality | | Respiratory mortality | |
| --- | --- | --- | --- | --- | --- | --- | --- |
|  |  | Percentage change | P-value | Percentage change | P-value | Percentage change | P-value |
| Urbanization level | 30.9% | -0.05 (-1.20 – 1.10) | 0.94 | -0.40 (-2.34 – 2.55) | 0.69 | -2.70 (-6.30 – 0.91) | 0.14 |
| Elderly | 2.6% | -0.11 (-1.25 – 1.03) | 0.85 | -0.10 (-2.11 – 1.92) | 0.92 | -1.14 (-4.94 – 2.67) | 0.56 |
| Comorbidities | 4.0% | 0.36 (-0.84 – 1.57) | 0.55 | 0.43 (-1.77 – 2.64) | 0.70 | 2.72 (-1.40 – 6.83) | 0.19 |
| Outdoor workers | 1.9% | 0.03 (-1.19 – 1.24) | 0.97 | 0.07 (-1.97 – 2.12) | 0.94 | 2.65 (-1.00 – 6.30) | 0.15 |
| Less than a high school education | 2.6% | 0.79 (-0.30 – 1.86) | 0.15 | -0.32 (-2.24 – 1.60) | 0.74 | 3.52 (0.48 – 6.57) | 0.07 |

**Figure S1.** Projected increase in temperature (°C, GCM-ensemble average) in 2090-99 compared to 2010-19 under SSP5-8.5 in 111 health regions.

**
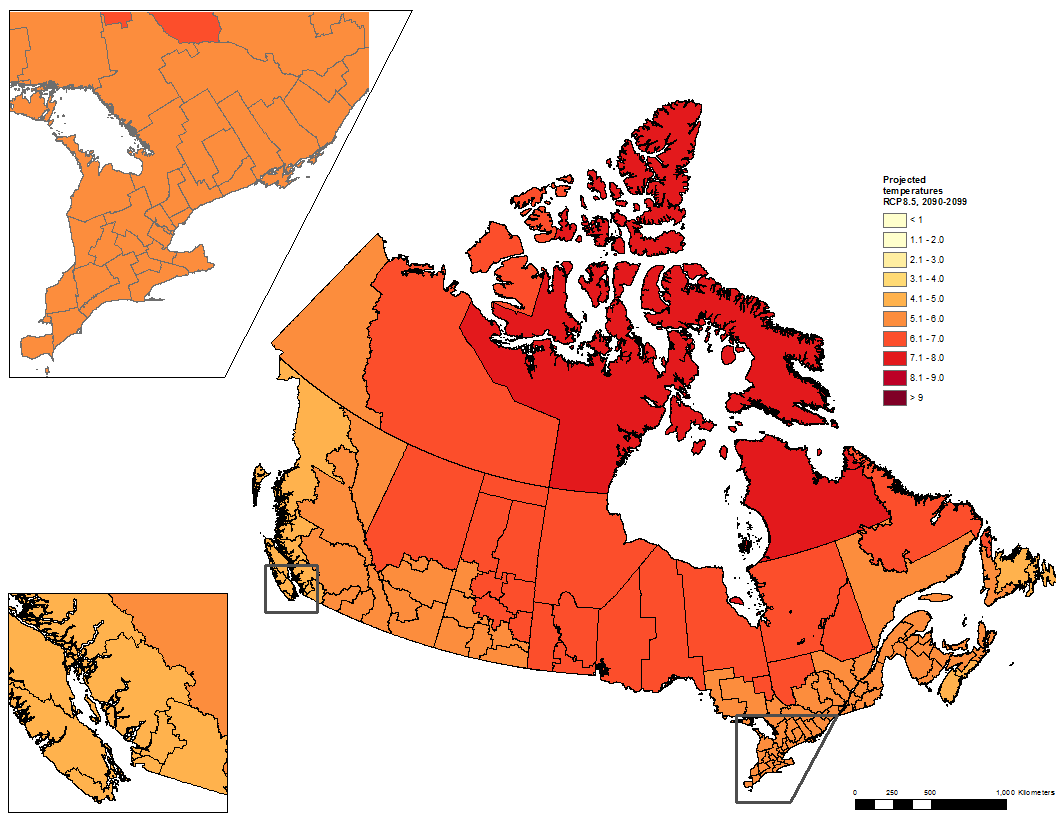
**
